# Health Risk-Based Assessment and Management of Heavy Metals-Contaminated Soil Sites in Taiwan

**DOI:** 10.3390/ijerph7103596

**Published:** 2010-10-11

**Authors:** Hung-Yu Lai, Zeng-Yei Hseu, Ting-Chien Chen, Bo-Ching Chen, Horng-Yuh Guo, Zueng-Sang Chen

**Affiliations:** 1 Department of Post-Modern Agriculture, MingDao University, Changhua 52345, Taiwan; E-Mails: soil.lai@mdu.edu.tw (H.-Y. Lai); bcchen@mdu.edu.tw (B.-C. Chen); 2 Department of Environmental Science and Engineering, National Pingtung University of Science and Technology, Pingtung 91201, Taiwan; E-Mails: zyhseu@mail.npust.edu.tw (Z.-Y. Hseu); chen5637@mail.npust.edu.tw (T.-C. Chen); 3 Division of Agricultural Chemistry, Taiwan Agricultural Research Institute, Council of Agriculture, Taichung 41301, Taiwan; E-Mail: hyguo@tari.gov.tw; 4 Department of Agricultural Chemistry, National Taiwan University, Taipei 10617, Taiwan

**Keywords:** risk-based assessment, heavy metal, soil remediation techniques, food safety risk, phytoremediation

## Abstract

Risk-based assessment is a way to evaluate the potential hazards of contaminated sites and is based on considering linkages between pollution sources, pathways, and receptors. These linkages can be broken by source reduction, pathway management, and modifying exposure of the receptors. In Taiwan, the Soil and Groundwater Pollution Remediation Act (SGWPR Act) uses one target regulation to evaluate the contamination status of soil and groundwater pollution. More than 600 sites contaminated with heavy metals (HMs) have been remediated and the costs of this process are always high. Besides using soil remediation techniques to remove contaminants from these sites, the selection of possible remediation methods to obtain rapid risk reduction is permissible and of increasing interest. This paper discusses previous soil remediation techniques applied to different sites in Taiwan and also clarified the differences of risk assessment before and after soil remediation obtained by applying different risk assessment models. This paper also includes many case studies on: (1) food safety risk assessment for brown rice growing in a HMs-contaminated site; (2) a tiered approach to health risk assessment for a contaminated site; (3) risk assessment for phytoremediation techniques applied in HMs-contaminated sites; and (4) soil remediation cost analysis for contaminated sites in Taiwan.

## 1. Introduction

### 1.1. Background of the Soil and Groundwater Pollution Remediation Act in Taiwan

Contaminated lands have been of concern in Taiwan for about three decades. Until now, soil remediation techniques and regulatory systems have been used to combat soil contamination, particularly for human and ecological health reasons. Because Taiwan is a highly industrialized country, and illegal waste water and solid waste disposal are the major sources of soil contamination. Among them, heavy metal (HM) contamination poses a major environmental and human health threat, and the cleanup of these soils continues to be time-costly and difficult. The specific form and reactivity of HMs and their association with soil components determine the ecotoxicological significance of their environmental impact [1].

During the 1980s, a crucial event of rice pollution by cadmium (Cd) occurred in Taoyuan County, in north-western Taiwan. Over 100 ha of paddy fields were contaminated by Cd and lead (Pb) by the illegal discharge of wastewater from a nearby plastic-stabilizer producing plant [2]. The farmers found the leaves of rice chlorotic and brown-dotted, and the whole plant died slowly before harvesting. Measurement of the soils and rice tissues revealed, shockingly, that the Cd concentration exceeded 10 mg/kg in the soils and 0.5 mg/kg in the rice grain at the sites, respectively. Since this Cd-rice pollution event, an increasing number of soils contaminated with HMs have been reported. These pollution events impacted the environmental regulation and food safety in Taiwan dramatically [3].

In order to fully address soil contamination in Taiwan, the Soil and Groundwater Pollution Remediation Act (SGWPR Act) was announced in 2000 [4]. There are eight chapters and 57 articles in the SGWPR Act. When the concentrations of contaminants exceed the soil control standard (SCS), this site will be announced as a “control site”. The polluter of a control site needs to take necessary actions to avoid further deterioration by contamination. Control sites assessed to have risk on human health by a tiered approach will be further designated as “remediation sites”. The remediation site cannot be sold and the polluter must remedy the site in accordance with the SGWPR Act.

### 1.2. One Target Regulation in Last Decade of the World

Soil control standards are often considered as trigger criteria to determine the need for a further soil investigation or remediation. These SCSs often have a different application in each legislative framework. In addition, their scientific basis and derivation procedures differ in various countries [5]. The variation in SCSs is the product of political consideration [6], risk perception [7], and differences in model parameters and algorithms with their boundary conditions. Provoost *et al.* [5] presented an example of the variation in SCSs in the Flemish region of Belgium, France, Germany, Great Britain, the Netherlands, Norway, Sweden, Switzerland, Canada, and USA for arsenic (As), Cd, chromium (Cr), copper (Cu), mercury (Hg), nickel (Ni), Pb, and zinc (Zn), for which the differences were more than two orders of magnitude.

Differences in clean-up standards in government regulations such as in or exclusion of ecotoxicological criteria in the derivation of the final SCS, differences in model parameters and algorithms with their boundary conditions, or the use of different bioconcentration factors for plant uptake have been compared [8]. Key elements involved in the legislative framework under one target regulation in a country therefore trigger remedial actions, which in turn trigger further soil investigation, differentiation between land use types, protection of human health, protection of the ecosystem, and protection of groundwater and surface water.

When data regarding soil contaminants are available, they should be compared with SCSs to reflect the maximum tolerable risk of exposure. However, such standards are not always in place. For instance, of the volatile organic carbon compounds detected in groundwater samples by the US Geological Service, 21 species were unregulated and without standards [9]. Patterson *et al.* [10] found a variety of brominated ethenes in Australian groundwater, for which there were no standards. When there are no standards, government policies tend to neglect the soil pollutant involved, which may lead to a divergence between risk as established by governments and actual risks.

### 1.3. Health Risk-Based Assessment Regulation

There is an increasing use of risk-oriented policies to deal with the local effects of soil contamination. A specific objective underlying the UK government’s approach to land contamination is to identify and remove unacceptable risks to human health and the environment [11]. Environmental risk assessment is a key element in appraisal so that decisions can be made on equitable policy and other factors.

In the UK, contaminated land is identified based on risk assessment. Part II of the Department of the Environment Transport and the Regions [11] section of the Environmental Protection Act, specifies that the source-pathway-receptor linkage concept for risk assessment is carried out to assess the risk of contaminated lands. The objective is to ensure the land is fit for either its current use or redevelopment. All three elements of the linkage must be present for a risk to exist. If any of the elements of a pollutant linkage is absent, there can be no risk and the land is deemed uncontaminated.

According to the SGWPR Act in Taiwan and for a control site, a control project can be conducted to decrease the health risk of contaminants to an acceptable level. At a remediation site, a risk assessment can be first processed by the pollution producer. The huge costs incurred by remediation sites during the process of remediation can be reduced enormously if the risk of exposure is controlled by suitable practices. When decreasing the concentrations of contaminants in a remediation site to below the SCS is not possible because of the limitation of geologic conditions, characteristics of the contaminant, and remediation techniques, risk assessment be carried out with the provision of a flexible remediation target. In addition, if the pollution producer is not identified, the director of the organization can consider financial and environmental factors in processing a risk assessment of the contaminated site.

## 2. Pollution Sources and Survey History of HMs Contaminated Sites in Taiwan

Over 100,000 industrial plants are currently registered in Taiwan, and approximately more than 20% of them undoubtedly produce hazardous wastewater. Taiwan’s government has obtained detailed information regarding solid waste disposal from 18,000 of these plants. Most plants generating wastewater and solid waste are located in more than 90 industrial parks administered by the government. However, about 50% of industrial parks offer a centralized system for wastewater collection and treatment. Approximately 30 wastewater treatment plants operate in the industrial parks, and the treatment capacity of the wastewater from 2,103 factories is 110,000 m^3^/day. However, 2,037 factories are responsible for treating their own wastewater (158,000 m^3^/day). Nevertheless, factories without registration outside the industrial parks were excluded in the above estimation of wastewater generation. Illegal discharge of swine wastewater caused the accumulation of nutrients (N, P, and soluble salts) and HMs (Cu and Zn) in nearby bodies of water. Paddy fields were consequently contaminated by this wastewater, which ran through the irrigation canal systems. Additionally, about 40% of the rivers in Taiwan have been contaminated by solid wastes and wastewater [12]. It is estimated that 30 million tons of industrial solid wastes are generated in Taiwan each year. These hazardous wastes comprise 10% of the total.

Rice (*Oryza sativa* L.), one of the most important cereal crops in the world, is the staple food in Taiwan. The total paddy field area is approximate 0.4 million ha, which is one-half of agricultural land in Taiwan. However, the paddy fields of Taiwan are threatened by a high potential for contamination. On the other hand, water resources for rice production in Taiwan have been contaminated by the illegal discharge of industrial and livestock wastewater, so that the paddy soil quality and rice safety have been adversely affected by HMs-contaminations [13,14]. Paddy fields are characterized by their submergence in water. The irrigation water may be contaminated by the above mentioned source of contamination [15]. As a result, the paddy soils have been contaminated not only by the HMs, but also by the residual HMs from the wastewater both of which are retained by sediments in the irrigation canal system. Over a long period, the increase of HM levels in the sediments also played a role as a contamination source of paddy soils in addition to the HMs directly transported into the rural soils from wastewater.

## 3. Exposure Assessment of the Contaminated Sites

Three transmission media that must be considered for calculating the risk of HMs, namely soil, groundwater, and air. The HMs may be ingested by humans from these pathways, which are listed as follows:

### 3.1. Exposure PATHWAys of Soil Medium

#### 3.1.1. Oral intake [16]

Oral intake of HMs from soil medium can be calculated by the following equation:

(1) $${\text{Intake}}_{\text{oral}-\text{soil}}=\frac{C_{\text{soil}}\times {\text{IR}}_{\text{oral}-\text{soil}}\times \text{EF}\times \text{ED}}{\text{BW}\times \text{AT}}\times \text{CF}$$

Intake_oral-soil_: exposure dose of oral intake (mg/kg/day)
C_soil_: concentration of concerned pollutant (mg/kg)
IR_oral-soil_: ingestion rate (mg/day)
EF: exposure frequency (day/year)
ED: exposure period (year)
BW: body weight (kg)
AT: average time (day)
CF: conversion factor (kg/mg)

#### 3.1.2. Dermal intake [17]

Dermal intake of HMs from soil medium can be calculated by the following equation:

(2) $${\text{Intake}}_{\text{dermal}-\text{soil}}=\frac{{\text{DA}}_{\text{event}}\times \text{EV}\times \text{ED}\times \text{SA}\times f_{\text{sa}}}{\text{BW}\times \text{AT}}$$

Intake_dermal-soil_: exposure dose of dermal intake (mg/kg/day)
DA_event_: exposure dose of each event (mg/cm^2^)
EV: event frequency (1/day)
SA: skin surface area (cm^2^)
f_sa_: ratio of surface of upper arm to body

(3) $${\text{DA}}_{\text{event}}={\text{C}}_{\text{soil}}\times \text{AF}\times {\text{ABS}}_{\text{d}}\times \text{F}$$

AF: adherence factor (mg/cm^2^)
ABS_d_: absorption factor

### 3.2. Exposure Pathways of Groundwater Medium

#### 3.2.1. Infiltration from soil to groundwater [18]

If the HMs downward moves and enters the groundwater, the consequent risk in groundwater depends on the concentration of HM presented in groundwater. If the concentration of a HM resulted from the downward movement (C_water_) is greater than the initial concentration in groundwater (C_i_), C_water_ is used to calculate the risk. The C_i_ is used on the contrary. For HMs, the C_water_ can be calculated using the following equation:

(4) $$C_{\text{water}}=C_{\text{soil}}\times \frac{1}{K_{d}}\times \frac{1}{\left(1+\frac{U_{\text{gw}}\times \delta_{\text{gw}}}{I\times W}\right)}$$

C_water_: concentration of concerned pollutant in groundwater (mg/L)
C_soil_: concentration of concerned pollutant in soil (mg/kg)
I: infiltration rate (cm/year)
K_d_: partition coefficient (cm^3^-water/g-soil)
W: maximum width between pollution source and groundwater (cm)
U_gw_: flow rate of groundwater (cm/year)
ä_gw_: height of mixing layer of groundwater (cm)

#### 3.2.2. Oral intake [16]

Oral intake of HMs from groundwater medium can be calculated by the following equation:

(5) $${\text{Intake}}_{\text{oral}-\text{water}}=\frac{{\text{C}}_{\text{water}}\times {\text{IR}}_{\text{oral}-\text{water}}\times \text{EF}\times \text{ED}}{\text{BW}\times \text{AT}}$$

Intake_oral-water_: exposure dose of oral intake (mg/kg/day)
C_water_: concentration of concerned pollutant in groundwater (mg/L)
IR_oral-water_: amount of drinking (L/day)

#### 3.2.3. Dermal intake [17]

Intake resulted from using contaminated groundwater during taking a bath or in regular washing and thus the contact with skin: the intake can be calculated by the following equation for HMs.

(6) $${\text{Intake}}_{\text{dermal}-\text{water}}=\frac{{\text{DA}}_{\text{event}}\times {\text{EV}}_{\text{shower}}\times \text{ED}\times \text{EF}\times \text{SA}}{\text{BW}\times \text{AT}}$$

Intake_dermal-water_: exposure dose of dermal intake (mg/kg/day)
DA_event_: exposure dose of each event (mg/cm^2^)
EV_shower_: frequency of each event (1/day)

### 3.3. Exposure Pathways of Air Medium [16,19]

Inhalant intake of HMs from air medium can be calculated by the following equation:

(7) $${\text{Intake}}_{\text{inh}-\text{soil}}=\frac{C_{\text{air}}\times {\text{IR}}_{\text{inh}}\times \text{EF}\times \text{ED}}{\text{BW}\times \text{AT}}$$

(8) $$C_{\text{air}}=C_{\text{soil}}\times \frac{P_{e}\times W}{U_{\text{air}}\times \delta_{\text{air}}}\times \text{CF}$$

Intake_inh-soil_: exposure dose of inhalant intake (mg/kg/day)
C_air_: concentration of HM in air (mg/m^3^)
C_soil_: concentration of HM in soil (mg/kg)
P_e_: particulate emission rate (g/cm^2^/sec)
W: maximum width of HM-contaminated site parallel with the wind (cm)
U_air_: wind speed above the HM-contaminated site (cm/sec)

### 3.4. Exposure of HM via Rice

Because HMs are absorbed by humans through the food chain from edible crops grown in contaminated soils, the concentrations of HMs in brown rice may be a critical problem for food safety herein. Rice is the staple food for daily consumption in Taiwan. Exposure via rice is therefore an important concern in the health of the population. The estimated daily intake (mg/kg/day BW) of HMs via rice consumption can be calculated as:

(9) $$\text{EDI}=\frac{C_{\text{on}}\times C\times \text{ED}\times \text{EF}}{\text{BW}\times \text{AT}}$$

where C_on_ (g/person/day) is the daily average consumption of brown rice in Taiwan, C (mg/kg) is the concentration of HMs in the contaminated rice, ED is exposure duration (70 years, equivalent to the average life), EF is exposure frequency (365 days/year), BW (kg/person) represents body weight, and AT is average time (365 days year/number of exposure years, assuming 70 years in this study). The average daily brown rice intake of adults and children was considered to be 100 and 50 g/person/day, respectively, and average adult and child body weights were considered to be 65 and 30 kg, respectively.

The regulation for Cd in brown rice in Taiwan is 0.4 mg/kg, while the upper limit of background Cd contents of representative rural soil is 3 mg/kg. If the total soil Cd content is higher than 5 mg/kg, most of the corresponding brown and polished rice is considered as Cd-contaminated rice [2].

Various rice cultivars have been cultivated in 19 Cd-contaminated paddy fields in Taiwan to evaluate the uptake of Cd [14]. The total soil Cd concentrations ranged from <0.1 mg/kg to almost 30 mg/kg, that is, background levels to heavily Cd-contaminated soils, respectively. In each field, 12 rice cultivars of Indica and Japonica varieties were planted on plots for each cultivar. The Cd concentrations in rice grains were quite different among rice varieties. The Indica species accumulated high concentrations of Cd in their rice grains [14]. For all Indica species, median levels of Cd in the rice grains exceeded the food quality standard (FQS) in the EU (0.2 mg/kg) as well as the FQS used in the WHO, Japan, and Taiwan (0.4 mg/kg). However, the Indica species was not suitable for cropping on paddy soils contaminated with Cd [20]. Cadmium contents in the rice grains of the Japonica species are lower than those of Indica species, although median Cd grain levels are close to or in excess of FQS in the EU. Even the total soil Cd levels below 0.3 mg/kg, a large number of rice grain samples of Indica and Japonica varieties did not meet the FQS. For Indica varieties, the percentage of samples in which Cd levels exceeded the FQS of 0.2 or 0.4 mg/kg was 51.1% and 11.3%, respectively. Hence, Indica varieties cannot be grown safely in Cd enriched soils without a considerable risk of exceeding the FQS in the EU. Moreover, the WHO set the provisional tolerable weekly intake of Cd for adult persons at 7 ìg/kg body weight/week [21], which means that the daily exposure via the abovementioned contaminated rice, will pose a serious risk for human health based on the calculation of Equation 9 in this paper.

The SCS of Cd (5 mg/kg) with *aqua regia* measurement for total analysis in the SGWPR Act in Taiwan was found unsuitable to assess the suitability or food safety for rice production. When the total soil Cd concentrations were below 5 mg/kg, 30% and 17 % of the grain samples of Japonica varieties did not meet the FQS of 0.2 and 0.4 mg/kg, respectively. For Indica varieties, these percentages increased to 41% for 0.4 mg/kg FQS to 73% for 0.2 mg/kg FQS [14]. These results clearly stressed the need to develop alternative soil testing methods or soil–plant uptake models to predict Cd uptake by rice grains in order to identify soils where rice can be grown safely [22].

### 3.5. Case Study of Exposure Assessment on HMs in Brown Rice of Different Varieties

The above case showed that the quality of rice did not meet the FQSs, which has affected consumer faith regarding the quality of rice. Many examples stress the need to predict the availability of HMs in soils in order to estimate the risk related to its uptake by crops. Due to differences in soil characteristics and pollutants in soil, the degree of availability is different, which has resulted in a wide range of soil quality standards.

Soil pH has a major influence on the availability of HMs that present predominantly as cations (Cu^2+^, Co^2+^, and Pb^2+^). Under acid conditions, sorption of HM cations by soil colloids is at a minimum, and the solution concentrations are relatively high. There is a relationship between the concentrations of HMs in the soil solution (HM_solution_) and those in the rice (HM_rice_). This relationship can be described in Equation 10.

(10) $$\text{Log}\;({\text{HM}}_{\text{rice}})={á}_{1}+{â}_{1}\times \text{log}\;({\text{HM}}_{\text{solution}})$$

The values of á_1_ and â_2_ can be derived from experimental data by linear regression after log transformation of the data. However, the concentration in soil solution is difficult to determine, so usually the total HM content was determined. There is a good relationship between soil properties and the HM content in the soil (HM_soil_) and the concentration in the soil solution (HM_solution_) [13]. If soil data of organic matter (OM), clay (the percentage <2 μm), and pH are available, these data can also be used to predict the uptake of HMs by rice.

(11) $$\text{Log}\;({\text{HM}}_{\text{solution}})={á}_{2}+{â}_{2}\times \text{log}\;({\text{HM}}_{\text{soil}})+{ã}_{1}\times \text{log}\;(\text{OM})+{ä}_{1}\times \text{log}\;(\text{clay})+{å}_{1}+\text{pH}$$

The values of á_2_, â_2_, ã_1_, ä_1_, and å_1_ can be derived from experimental data by linear regression after log transformation of the data. The addition “2” indicates that á and â in Equation (11) differ from those in Equation (10). The last two Equations, 10 and 11, can be combined in Equation (12) to predict the uptake by rice directly from soil properties without measuring the soil solution [14].

(12) $$\text{Log}\;({\text{HM}}_{\text{rice}})={á}_{3}+{â}_{3}\times \text{log}\;({\text{HM}}_{\text{soil}})+{ã}_{2}\times \text{log}\;(\text{OM})+{ä}_{2}\times \text{log}\;(\text{clay})+{å}_{2}+\text{pH}$$

The values of á_3_, â_3_, ã_2_, ä_2_, and å_2_ in Equation (12) can be derived from the combination of Equations (10) and (11) and they will be different from those in Equations (10) and (11). Although some models clearly need to be improved, experimentally derived models were used to calculate the risk for rice cropping. This calculation was done by calculating the critical levels of Cd in the soil above which the Cd content of brown rice exceeds the FQS.

## 4. Health Risk-Based Assessment of the Contaminated Sites

### 4.1. Models for Health Risk-Based Assessment

Hazard identification, hazard assessment, risk estimation, and risk evaluation are the four stages in risk assessment. Risk characterization is performed to calculate the risk after the determination of the exposure dose from different pathways. The carcinogenic risk (R_total_) and the non-carcinogenic risk are used to indicate the risk from oral intake (R_oral_), inhalation (R_inh_), and skin contact (R_dermal_). The carcinogenic risk is the sum of these three individual risks, and they are calculated in Equations 13–15. A total carcinogenic risk equal to or less than 10^−6^ is acceptable.

(13) $${\text{R}}_{\text{oral}}=({\text{Intake}}_{\text{oral}-\text{water}}+{\text{Intake}}_{\text{oral}-\text{soil}})\times {\text{SF}}_{\text{oral}}$$

(14) $${\text{R}}_{\text{inh}}=({\text{Intake}}_{\text{inh}-\text{water}\;(\text{total})}+{\text{Intake}}_{\text{inh}-\text{soil}\;(\text{total})})\times {\text{SF}}_{\text{inh}}$$

(15) $${\text{R}}_{\text{dermal}}=({\text{Intake}}_{\text{dermal}-\text{water}}+{\text{Intake}}_{\text{dermal}-\text{soil}})\times {\text{SF}}_{\text{dermal}}$$

SF_oral_, SF_inh_, and SF_dermal_ are carcinogenic slopes of intake HM from the oral pathway, inhalant pathway, and dermal pathway, respectively.

Non-carcinogenic risk (hazard quotient, HQ) is determined by using the hazard index (HI), which is the sum of HQ from the three pathways. The acceptable HI value is equal to or less than unity and can calculated using Equation 16.

(16) $$\text{HI}={Ó\text{HQ}}_{\text{oral}}+{Ó\text{HQ}}_{\text{inh}}+{Ó\text{HQ}}_{\text{dermal}}$$

(17) $${\text{HQ}}_{\text{oral}}=\frac{\left({\text{Intake}}_{\text{oral}-\text{water}}+{\text{Intake}}_{\text{oral}-\text{water}}\right)}{{\text{RfD}}_{\text{oral}}}$$

(18) $${\text{HQ}}_{\text{inh}}=\frac{\left({\text{Intake}}_{\text{inh}-\text{water}\;(\text{total})}+{\text{Intake}}_{\text{inh}-\text{water}\;(\text{total})}\right)}{{\text{RfD}}_{\text{inh}}}$$

(19) $${\text{HQ}}_{\text{dermal}}=\frac{\left({\text{Intake}}_{\text{dermal}-\text{water}}+{\text{Intake}}_{\text{dermal}-\text{water}}\right)}{{\text{RfD}}_{\text{dermal}}}$$

RfD_oral_, RfD_inh_, and RfD_dermal_ are the reference dose of a non-carcinogenic pollutant from oral pathway, inhalant pathway, and dermal pathway, respectively.

Many risk assessment models are used to quantify exposure to a contaminant and to determine the generic or site-specific assessment criteria (Table 1).

### 4.2. Case Study in Tiered Approach Health Risk Assessment for a Contaminated Site

#### 4.2.1. Site description

A remediation site in southern Taiwan was used in a case study of the tiered approach in health risk assessment. This site had been contaminated by illegal dumping of hazardous wastes including illicit business backfill sludge, flue dust, and construction waste. The hazardous wastes were removed from the site; however, residual HMs left at the site caused concern. The site length from north to south is about 130 m and its width from west to east is about 50 m. Its surroundings are close to neighboring paddy fields, factories, fishing ponds, and residential areas. The area of the contaminated site is approximate 5,900 m^2^. In this case study, the spatial variation of HM concentration was investigated by using a grid sampling method. The soil samples were obtained from the depths of 0–15, 15–30, and 30–45 cm. The concentrations of HMs (As, Cd, Cr, Cu, Hg, Ni, Pb, and Zn) after aqua regia digestion were determined by either atomic absorption spectrometer (AAS) or inductively coupled plasma-mass spectrometry (ICP-MS). The soil analytical results showed that the average concentrations of Cr, Cu, Ni, Pb, and Zn were over the SCSs for non-farmlands in Taiwan (Table 2). However, the concentrations of the HMs varied greatly at the site.

#### 4.2.2. Processes of tier risk assessment

In first tier risk assessment, the concentration of HM was involved in the case of exposure assessment for both adults and children living near residential and industrial areas via the three main exposure pathways: (1) direct ingestion of soil substrate particles; (2) dermal absorption of HMs in particles adhered to exposed skin; and (3) inhalation of suspended particles through the mouth and nose. The exposure dosage through above pathways considered was calculated by Equations 1–3 and Equations 7–8 [24]. In second tier risk assessment, the involved parameter was initialized by the 95 percent upper confidence limit (95% UCL) of mean concentrations for the HMs listed in Table 2, and the other processes in exposure scenarios, receptors, and exposure pathways were the same as those used in the first tier risk assessment considered. The 95% UCL was followed Equation (20) for individual HM. Third tier risk assessment used a probabilistic approach for the HM concentration (Table 2) in addition to some exposure parameters. Other processes of exposure scenarios, receptors, and exposure pathways were the same as those used in tier 1 and tier 2 risk assessments were considered.

(20) $$95\%\text{UCL}=\bar{x}+t_{0.95,n-1}(\frac{s}{\sqrt{n}})$$

where *_x̄_* is sample mean, *n* is sample size, *s* is sample standard deviation, and *t_0.95, n-1_* is *t* critical value at 95% confidence interval and *n-1* degree of freedom.

#### 4.2.3. Results of tier risk assessment

The tiered approach health risk assessment results are listed in Table 3. The first tier risk assessment indicated that the carcinogenic risk and the non-carcinogenic risk were 8.2 × 10^−5^ to 1.7 × 10^−4^ and 57.0 to 88.2, respectively, which were clearly higher than the acceptable standard 1 × 10^−6^ and 1.0, respectively. The maximum of Cr concentration at the contaminated site was 20,700 mg/kg. This value is very high, and Cr appeared to be the largest single contributor to the overall risk, which for carcinogenic risk ranged from 7.0 × 10^−5^ to 9.4 × 10^−5^ for adults and 1.5 × 10^−4^ for children.

The highest contribution to the overall risk by exposure pathway was ingestion of soil particles (78%), followed by inhalation of the HMs in these particles (17%). Intake of Cr may cause lung tumors; mortality increases through inhalation exposure, and causes several internal organ cancers through ingestion [25]. Chromium in the non-carcinogenic risk is also the largest single contributor with a hazard quotient (HQ) of 55 and 78 in adults and children, respectively. Nickel is the second contributor after Cr, followed by Cu, Zn, As, and Hg. The highest contribution to the overall risk by exposure pathway was dermal absorption of the HMs followed by ingestion of soil particles.

The second tier risk assessment shows that the carcinogenic risk ranged from 1.2 × 10^−5^ to 1.7 × 10^−5^ for adults and 2.3 × 10^−5^ for children; both risks were greater than the standard 1.0 × 10^−6^. Arsenic is the largest contributor to the overall risk, and Cr is the second largest contributor followed by Ni. The exposure pathway that has the highest contribution to the overall figure of risk appears to be ingestion (>71%), followed by inhalation and dermal absorption of HMs in soil particles. The second tier risk assessment shows that the non-carcinogenic risk is 3.8 for adults and 6.1 for children; both risks were also greater than the standard 1.0, and deemed as unacceptable by the regulatory agency. Both exposure pathways of dermal contact and ingestion were the primary contributors to the overall risk.

The comparison of tier 2, which uses the 95% UCL of mean with lognormal distribution to calculate risk, with tier 1, which uses the maximum concentration, shows that the average carcinogenic risk reduces by 85%, and the average non-carcinogenic risk reduces by 93%. The risk value difference comes from the non-uniform distribution of the concentration while supporting the use of logarithm mean value may obtain good results. However, the concentration of contaminants in the soil will migrate over time with different mobility rates, so there is uncertainty [26].

The third tier risk assessment with lognormal distribution for target HMs and normal distribution of some parameters shows that the carcinogenic risk ranged from 8.6 × 10^−6^ to 1.1 × 10^−5^ for adults and 2.0 × 10^−5^ for children at median and 2.9 × 10^−5^ to 3.5 × 10^−5^ for adults and 8.8 × 10^−5^ for children at 95 percentile. The non-carcinogenic risk ranged from 1.9 to 2.1 for adults and 6.0 for children at median and 17 to 18 for adults and 37 for children at 95 percentile; both risks were greater than standard 1.0. Comparing the results of tier 3 risk assessment shows that the median values of both carcinogenic and non-carcinogenic risks were lower than results obtained from tier 2 assessment with 95% UCL. The 95 percentile risks in tier 3 assessment were lower than those of tier 1 assessment with maximum HM concentrations for which the average reductions of risk were 60% and 65% for carcinogenic and non-carcinogenic risks, respectively. The relatively high risk in tier 1 assessment indicated that the risk assessment could be overestimated with maximum HM concentrations.

The quantitative estimation of risk in this study is also affected by a high degree of uncertainty in the estimates of exposure rates and is probably more significant than the toxicity data used in the risk assessment. They are being reviewed permanently with considerable changes in their values and sometimes even in the threshold or non-threshold behavior of the toxicant. All these considerations suggest that the impact of risk assessment of this or similar studies should be carefully analyzed.

### 4.3. Case Study of Risk Assessment for a Contaminated Site before and after Remediation with an Attenuation Method

This case study examined the concentration variation and health risk assessment for a HMs-contaminated farmland before and after treatment using an attenuation mixed method. The attenuation method is a dilution method, which in the contaminated site the slightly HMs contaminated and uncontaminated soil were used to mix high HMs contaminated soil in site. After the soil mixed the HMs concentrations of soil mixture in contaminated soil are below SCSs. Concentrations of HMs in the soil lacked homogeneous variance and non-normal distribution, so the HM concentration distribution was fitted to lognormal distribution to obtain a 95% UCL and concentration probability distributions.

#### 4.3.1. Site description and soil analysis

The 27.72 ha study site is located in Changhua County, Central Taiwan. It is a farmland polluted by electroplating wastewater. Before treatment, the survey data showed that the concentration of HMs was highest in the irrigation intake inlet while concentrations decreased with distance away from the inlet. HMs concentrations decreased with increasing soil depth and were focused within 60 cm layer of the ploughed layer. The soil below the ploughed layer was considered the source of attenuation in a vertical direction.

Soil sampling was divided into four layers with depths of 0–15, 15–30, 30–45, and 45–60 cm, respectively. Sampling size was dependent on the polluted condition of the soil. Highly polluted soil was sampled more extensively according to the survey data. The soil sampling procedure and method followed the Taiwan EPA (Taiwan Environmental Protection Administration) standard method. Quality assurance and quality control (QA/QC) were carried out according to Taiwan EPA standard procedures. The samples were digested by aqua regia, and then the concentrations of HMs (Cr, Cu, Ni, and Zn) were determined by either AAS or ICP-MS. The soil analytical results showed that the maximum concentrations of Cr, Cu, Ni, and Zn before and after soil dilution remediation procedure were over the SCS for non-farmlands (Table 4). However, the concentrations of the HMs varied greatly before the treatment at the site.

#### 4.3.2. Risk assessment and analyses

In this study, the hazardous substances were four HMs, namely Cr, Cu, Ni and Zn. Toxicology factors, cancer slope factors (SF) for carcinogenic risk, and reference dose (RfD) for non-carcinogenic risk were taken from the database in the Integrated Risk Information System, which is built by USEPA [25]. Exposure assessments for both adults and children living in the nearby residential area and adults working in the nearby industrial area were considered via three exposure pathways, which included ingestion of HM-polluted soil, dermal absorption by contacting HM-polluted soil, and inhalation of soil particulates containing HM. Calculations of exposure doses was carried out by Equations 1–3 and 7–8. Three HMs concentration tiers were calculated as the maximum values for tier 1, 95% UCL for tier 2, and the probability distribution of concentration for tier 3. The carcinogenic risk assessment was carried out by Equations 13–15 and the non-carcinogenic risk assessment was carried out by Equations 17–19.

#### 4.3.3. Comparison of risk assessment between before and after soil remediation

The health risk assessment results for the tiered approach are listed in Table 5. Soil attenuation remediation before the first tier risk assessment indicated that the carcinogenic risk and the non-carcinogenic risk were 6.1 × 10^−6^ to 1.5 × 10^−5^ and 6.4 to 11.0, respectively, which were much higher than that of the acceptable standard. After soil attenuation remediation, the first tier risk assessment indicated that the carcinogenic risk and the non-carcinogenic risk were 2.0 × 10^−6^ to 4.9 × 10^−6^ and 2.1 to 3.2, respectively, which were still higher than the acceptable standard. However, after soil remediation treatment, the health risk was reduced by 67% and by 69% for carcinogenic and the non-carcinogenic risks, respectively. The maximum of Cr concentrations at the contaminated site were 2,225 and 750 mg/kg before and after treatment, respectively, which showed that Cr is the largest single pollution contributor to the overall health risk. The highest contributions by exposure pathways to the carcinogenic and non-carcinogenic risks were ingestion of soil particles and dermal absorption of the Cr, respectively.

Before the soil remediation treatment, the second tier risk assessment showed that the carcinogenic risk ranged from 0.46 × 10^−6^ to 0.62 × 10^−6^ for adults and 1.1 × 10^−6^ for children. The risk to children is still greater, and Cr is the largest contributor. The exposure pathway that has the highest contribution to the overall risk value appears to be the ingestion of HMs in these particles. The second tier risk assessment showed that the non-carcinogenic risk ranged from 0.49 to 0.54 for adults and 0.84 for children. Both dermal and ingestion exposure pathways were the primarily contributors to the overall risk assessment.

The comparison between the tier 2 approaches using 95% UCL of mean value and the tier 1 approach using maximum concentration to calculate risk showed that the average carcinogenic risk assessment was reduced by about 93% and 87% before and after the soil remediation treatment, respectively. The average non-carcinogenic risk assessment was reduced by about 92% and 87% before and after soil remediation treatment, respectively. The differences between two risk assessments values derive from the spatial distribution of soil pollutants at the site, which are non-uniform. The minor soil samples have extremely high concentration; supporting the use of logarithm mean value may obtain good results.

The third tier risk assessment with lognormal distribution for target HMs and normal distribution of some parameters showed that the carcinogenic risk ranged from 0.19 × 10^−6^ to 0.23 × 10^−6^ for adults and 0.6 × 10^−6^ for children for the contaminated site before soil remediation. After soil remediation, it ranged from 0.14 × 10^−6^ to 0.18 × 10^−6^ for adults and 0.45 × 10^−6^ for children for median value of HMs. Both risk assessments before and after remediation were lower than that of standard value 1.0 × 10^−6^. The non-carcinogenic risk ranged from 0.23 to 0.25 for adults and 0.62 for children for contamination site before soil remediation and ranged from 0.16 to 0.17 for adults and 0.38 for children for median value of HMs after soil remediation. Both risk assessments before and after soil remediation were lower than standard value of 1.0.

The carcinogenic risk ranged from 1.0 × 10^−6^ to 1.3 × 10^−6^ for adults and 3.7 × 10^−6^ for children before treatment. Both risks were greater than the standard 1.0 × 10^−6^, and 0.48 × 10^−6^ to 0.6 × 10^−6^ for adults and 1.8 × 10^−6^ for children after treatment. After treatment, the carcinogenic risk was lower than the standard for adult but was still higher than the standard for children in the worst condition (95%). However, 84% of the carcinogenic risk is lower than the standard. In addition, comparison of the values before and after treatment showed that the carcinogenic risk decreased in average by 52%. The non-carcinogenic risk ranged from 0.95 to 0.97 for adults and 2.1 for children before treatment, and 0.43 to 0.45 for adults and 0.95 for children after treatment. After treatment, the non-carcinogenic risk was lower than standard 1.0 for both adults and children. The non-carcinogenic risk decreased on average by 55% in comparison of the values after and before treatment. Comparison of the results of tier 3, the median values for both carcinogenic and non-carcinogenic risks were lower than results obtained from tier 2 with 95% UCL. The 95 percentile risks in the tier 3 assessment were much lower than the results of the tier 1 assessment with maximum HM concentrations. The average reductions of risk were 87% and 84% for carcinogenic and non-carcinogenic risks before treatment, respectively, and the average reductions of risk were 72% and 77% for carcinogenic and non-carcinogenic risks after treatment, respectively. The relatively high risk in tier 1 assessment indicated that the risk assessment could be overestimated with maximum HM concentrations. The soil attenuation method applied to HM-contaminated farmland showed that the concentrations of HMs were uniform in each layer and risks were reduced significantly after treatment, which indicates that the soil attenuation method is suitable for application to slightly or moderately HMs-contaminated farmland.

### 4.4. Case Study of Phytoremediation Techniques for HMs-Contamination Soils

More than 200 ha of HMs-contaminated soils were found in central Taiwan; they were contaminated mainly with Cr, Cu, Ni, and Zn produced by irrigation with HMs-contaminated water discharged by surrounding electroplating plants. A large-area experiment with a total area of 1.3 ha supported by the Taiwan EPA was conducted from 2005 to 2006 to assess the feasibility of phytoremediation by planting 12 species. Soil samples of topsoil (0–15 cm) and subsoil (15–30 cm) were collected and analyzed for the total concentration of eight HMs (As, Cd, Cr, Cu, Hg, Pb, Ni, and Zn). The studied site was mainly contaminated by Cr, Cu, Ni, and Zn and some of the concentrations were much higher than the soil regulation for HMs in soil (Table 6). The median and maximum concentration of Cr, Cu, Ni, and Zn of this site were used for risk assessment by calculating carcinogenic and non-carcinogenic risks.

The method used for risk assessment included hazard identification, exposure assessment, dose-response assessment, and risk characterization [27]. Because some concentrations of the Cr, Cu, Ni, and Zn were higher than that of SMS (soil monitoring standard) or SCS, they were used as the target contaminants. Among them, Cu and Zn are non-carcinogenic chemicals and Cr and Ni are carcinogenic chemicals [24].

Exposure assessment was used to assess the total dose entering the human body through different pathways; its unit was always expressed as mg/kg/day. The median and maximum concentrations of Cr, Cu, Ni, and Zn in the topsoil were used in this study to calculate the mean and maximum effect of contaminants (Table 7).

Equations 21–23 were used to calculate the exposure risk resulting from the ingestion of contaminated soils (EXP_ing_), inhalation of air containing contaminated soil particles (EXP_inh_), and absorption by skin (EXP_abs_), respectively [24]. A mean body weight (60.9 kg) was used according to Jang *et al.* [28] and the supposed period of carcinogenic and non-carcinogenic risk was 70 and 30 years, respectively [24]:

(21) $${\text{EXP}}_{\text{ing}}=\frac{\text{CS}\times \text{IR}\times \text{EF}\times \text{ED}\times \text{CF}}{\text{BW}\times \text{AT}}$$

(22) $${\text{EXP}}_{\text{inh}}=\frac{\text{CS}\times \text{PA}\times \text{IH}\times \text{EF}\times \text{ED}\times \text{CF}}{\text{BW}\times \text{AT}}$$

(23) $${\text{EXP}}_{\text{abs}}=\frac{\text{CS}\times \text{AF}\times \text{ABS}\times \text{SA}\times \text{EF}\times \text{ED}\times \text{CF}\times \text{EV}}{\text{BW}\times \text{AT}}$$

Carcinogenic and non-carcinogenic effects were divided into dose-response assessment that used SF and RfD, respectively. In this study, the values of RfD and SF were in accordance with the value [27] and modified by Equation 24 because the average body weight of the exposure population of this study is 60.9 kg. The carcinogenic risk can only be calculated for inhalation because there were no SF values for the other two pathways.

(24) $$\text{Modified}\;\text{values}=\text{standard}\;\text{values}\;\times {\left(\frac{\text{BW}}{70}\right)}^{\frac{1}{3}}$$

Equation 25 was used to calculate the non-carcinogenic risk of contaminants to the health of humans [29], where HQ is the hazard quotient and EXP_total_ is the sum of total exposure. There are non-carcinogenic risks when the values of HQ are greater than unitary. The carcinogenic risk can be calculated by using Equation 26 and there are no carcinogenic risks when the TR values are less than 10^−6^ [29]. The results showed that although the study site was contaminated with HMs, the calculated values of HQ and TR for this site were less than unity and 10^−6^, respectively (Tables 8, 9). Therefore, there are no carcinogenic and non-carcinogenic risks although some of the total concentrations of Cr, Cu, Ni, and Zn were higher than the SMSs or SCSs.

(25) $$\text{HQ}={\text{EXP}}_{\text{total}}/\text{RfD}$$

(26) TR=EXP×SF

## 5. Remediation Cost Analysis of Case Studies in Taiwan

Once contaminants have entered the soil system, it is difficult to clean up the soil because of the complex reactions between soils and contaminants. The cost of remedying a control site or a remediation site that has been contaminated with petroleum or HM is always more than US$ 0.1 million, and at some sites the cost could reach several million US$. The huge cost of remediation usually makes it difficult to put in operation. According to the SGWPR Act in Taiwan, a suitable target value concentration based on risk assessment can be accepted in place of the SCS. There will be more selectivity in techniques, and the cost will decrease when the risk-based target concentration is used for control sites or remediation sites.

To remediate HMs-contaminated sites, costs were incurred by many items including site survey, remediation practices, soil fertility recovery, and environmental monitoring. According to previous projects the cost for soil turnover and mixing to dilute the concentration of HMs in each cubic meter of contaminated soils is estimated at US$ 10. Thermal treatment or landfill of one cubic meter of contaminated soils costs US$ 100 to 300 depending on the pollution status [30]. If the contaminated soils were excavated and transported to produce light density materials, the cost is usually less than US$ 100 for each cubic meter of contaminated soils. For instance, there were about 800 m^2^ contaminated with Cu, Ni, Pb, and Zn in central Taiwan resulting from the unsuitable disposal of sewage sludge. The contaminated soil was to a depth of 0.5 to 1.5 m and the total volume of the contaminated soil was approximately 5,480 m^3^. Soil turnover and dilution remediation method was applied to this site to remove the pollutants (3,700 m^3^), the concentration of which was 1 to 2.5-fold of soil regulation for HMs. About 380 m^3^ of contaminated soils, in which the concentrations of HMs in the soils that were 2.5 to 10-fold of soil regulation or SCS values, were excavated and transported to produce light density materials for other uses. Highly contaminated sites—about 1,400 m^3^ soil with a high concentration of HMs, which is 10-fold of SCS values—were excavated and transported to suitable landfill sites. The total cost to remediate this site to meet the values of SCS is estimated at US$ 420,000.

## 6. Conclusions

Many HMs-contaminated sites have been found in Taiwan during the last two decades resulting mainly from irrigation systems contaminated by wastewater illegally discharged from industrial parks located in the western regions of Taiwan. In some cases, it is difficult to remove the HMs from the soils due to local environmental limitations and very high remediation costs. According to the SGWPR Act of Taiwan, it is acceptable for the contamination producer to use suitable soil remediation techniques based on risk assessment to reduce the health risk instead of a total remediation process. However, limited studies have been conducted on risk assessment in Taiwan due to the lack of experience in soil remediation. One target regulation seems to be unfeasible because of the variation among sites. However, the reduction of the health risks of a contaminated site to an acceptable level is more important because the final objective of the SGWPR Act is to protect the health of the public.

## Figures and Tables

**Table 1.** Risk assessment tools [23].

**Tool^a^**	**Receptor**	**Use**	**Medium**
CLER	People	Develop UK soil guideline values	Compiled software
SNIFFER	People	Develop site-specific assessment criteria	Spreadsheet and paper worksheets
RBCA	People and groundwater	Determine site-specific assessment criteria	Programmed spreadsheet
BP RISC	People and groundwater	Determine site-specific assessment criteria	Compiled probabilistic or deterministic software

a CLER: Computer Lab for Experimental Research; SNIFFER: Scotland & Northern Ireland Forum for Environmental Research; RBCA: Risk-Based Corrective Action; BP RISC: British Petroleum Risk Integrated Software for Cleanups.

**Table 2.** Summary statistics of the analytical results of HMs concentrations at contaminated sites (mg/kg, n = 22).

**Element**	**Max.**	**95% UCLa**	**Lognormal distribution**	**Soil control standards in Taiwan**	
				**Non-farmland Farmland**	
As	14.80	9.44	8.85 b, 2.85 c	60	--
Cd	25.30	2.90	4.29, 11.0	20	5
Cr	20,700	1339	2294, 6369	250	--
Cu	9,080	863	1460, 3957	400	200
Hg	3.34	0.24	0.31, 0.56	20	5
Ni	12,100	764	1278, 3401	200	--
Pb	13,100	1266	2795, 11082	2000	500
Zn	26,300	1749	2983, 8175	2000	600

a UCL: upper confidence limit;

b mean;

c standard deviation

**Table 3.** Comparison of the tired approach risk assessment between carcinogenic risk and non-carcinogenic risk.

**Risk assessment**	**Exposure scenario**	**Receptor**	**Tier 1 Max.**	**Tier 2 95% UCL**	**Tier 3 Probability 50%, 95%**
Carcinogenic risk	Residential	Adults	1.1 × 10^−4^	1.70 × 10^−5^	(1.1 a, 3.5 b) × 10^−5^
	Residential	Children	1.7 × 10^−4^	2.3 × 10^−5^	(2.0, 8.8) × 10^−5^
	Industrial	Adults	8.2 × 10^−5^	1.2 × 10^−5^	(8.6, 29) × 10^−6^

Non-carcinogenic risk	Residential	Adults	57.0	3.8	1.9, 17
	Residential	Children	88.2	6.1	6.0, 37
	Industrial	Adults	57.0	3.8	2.1, 18

a 50 percentile;

b 95 percentile

**Table 4.** Summary statistics of the analytical results of HMs concentrations at contaminated sites (mg/kg).

**Element**	**Max.**	**95% UCL**	**Lognormal distribution**	**Soil control standards in Taiwan**	
				**Non-farmland Farmland**	
Cr (n = 894)^a^	2,225	171	153 c, 187 d	250	--
Cr (n = 372)^b^	750	96	88.2, 56.1	250	--
Cu (n = 889)	1,921	153	133, 156	400	200
Cu (n = 499)	737	64.5	59.4, 37.6	400	200
Ni (n = 885)	3,633	247	218, 268	200	--
Ni (n = 672)	309	93.2	88.4, 45.0	200	--
Zn (n = 863)	4,529	541	484, 542	2,000	600
Zn (n = 445)	1,370	242	227, 142	2,000	600

a before treatment,

b after treatment,

c mean,

d standard deviation

**Table 5.** Comparison of the tiered approach risk assessment between carcinogenic risk and non-carcinogenic risk before and after soil remediation treatment.

**Risk assessment**	**Exposure scenario**	**Receptor**	**Tier 1 Max.**	**Tier 2 95% UCL**	**Tier 3 Probability 50%, 95%**
Carcinogenic risk	Residential	adults (before) ‡	8.2 × 10^−6^	0.62 × 10^−6^	(0.23^a^, 1.3^b^) × 10^−6^
	
		adults (after) ^‡^	2.7 × 10^−6^	0.35 × 10^−6^	(0.18^a^, 0.6^b^) × 10^−6^
	Residential	children (before)	15.0 × 10^−6^	1.1 × 10^−6^	(0.6, 3.7) × 10^−6^
		children (after)	4.9 × 10^−6^	0.63 × 10^−6^	(0.45, 1.8) × 10^−6^
	Industrial	adults (before)	6.1 × 10^−6^	0.46 × 10^−6^	(0.19, 1.0) × 10^−6^
		adult (after)	2.0 × 10^−6^	0.26 × 10^−6^	(0.14, 0.48) × 10^−6^

Non-carcinogenic risk	Residential	adults (before)	7.1	0.54	(0.25 a, 0.95 b)
	
		adults (after)	2.3	0.3	(0.17 a, 0.43 b)
	Residential	children (before)	11.0	0.84	(0.62, 2.1)
		children (after)	3.2	0.43	(0.38, 0.95)
	Industrial	adults (before)	6.4	0.49	(0.23, 0.97)
		adults (after)	2.1	0.27	(0.16, 0.45)

‡: before: before soil remediation; after: after soil remediation.

a 50 percentile;

b 95 percentile

**Table 6.** The total concentration of HMs in the topsoil and subsoil of the site (mg/kg).

	**Total concentration of HMs in soil#**			

**HMs**	**SCS**	**SMS**	**Topsoil (0–15 cm)**	**Subsoil (15–30 cm)**
Cr	250 *	175 *	46 ∼ 463	38 ∼ 236
Cu	200	120	23 ∼ 152	26 ∼ 153
Ni	200 *	130 *	103 ∼ 523	48 ∼ 422
Zn	600	260	202 ∼ 958	139 ∼ 722

# SCS: soil control standard; SMS: soil monitoring standard

* According to SGWPR Act, there are only standards for non-farmlands

**Table 7.** The median and maximum concentrations of Cr, Cu, Ni, and Zn in topsoil (mg/kg).

**HMs**	**Total concentration of HMs in soil**	
	**Median value**	**Maximum value**
Cr	83	207
Cu	90	122
Ni	219	412
Zn	346	662

**Table 8.** The hazard quotient (HQ) and the sum of total exposure (EXP_total_) of the three pathways.

	**Zn**		**Cr**		**Cu**		**Ni**	

	**Med.**	**Max.**	**Med.**	**Max.**	**Med.**	**Max.**	**Med.**	**Max.**
EXP_inh_	5.9 × 10^−6^	1.1 × 10^−5^	1.4 × 10^−6^	3.5 × 10^−6^	1.5 × 10^−6^	2.1 × 10^−6^	3.7 × 10^−6^	7.0 × 10^−6^
EXP_ing_	2.7 × 10^−4^	5.2 × 10^−4^	6.5 × 10^−5^	1.6 × 10^−4^	7.1 × 10^−5^	9.6 × 10^−5^	1.7 × 10^−4^	3.2 × 10^−4^
EXP_abs_	4.7 × 10^−5^	8.9 × 10^−5^	1.1 × 10^−5^	2.8 × 10^−5^	1.2 × 10^−5^	1.6 × 10^−5^	3.0 × 10^−5^	5.6 × 10^−5^
HQ	1.0 × 10^−3^	2.0 × 10^−3^	2.5 × 10^−2^	6.2 × 10^−2^	3.0 × 10^−3^	4.1 × 10^−3^	9.8 × 10^−3^	1.9 × 10^−2^

Med.: median value; Max.: maximum value

**Table 9.** The exposure and carcinogenic risk if only the inhalation was considered.

	**Cr**		**Ni**	

	**Med.**	**Max.**	**Med.**	**Max.**
EXP_inh_ (mg/kg/day)	2.1 × 10^−8^	5.1 × 10^−8^	5.3 × 10^−8^	1.0 × 10^−7^
TR	7.0 × 10^−7^	1.8 × 10^−6^	3.7 × 10^−8^	7.0 × 10^−8^

Med.: median value; Max.: maximum value

## References

[1] He ZL Yang XE Stoffella PJ Trace elements in agroecosystems and impacts on the environment J. Trace Elem. Med. Bio 2005 19 125 140 16325528 10.1016/j.jtemb.2005.02.010

[2] Chen ZS Cadmium and lead contamination of soils near plastic stabilizing materials producing plants in northern Taiwan Water Air Soil Poll 1991 57–58 745 754

[3] Hseu ZY Su SW Lai HY Guo HY Chen TC Chen ZS Remediation techniques and heavy metals uptake by different rice varieties in heavy metals-contaminated soils of Taiwan: New aspects for food safety regulation and sustainable agriculture Soil Sci. Plant Nutr 2010 56 31 52

[4] Taiwan EPA Soil and Groundwater Pollution Remediation Web Available online: http://sgw.epa.gov.tw/public/En/index.htm (accessed on 30 June 2010).

[5] Provoost J Cornelis C Swarties F Comparison of soil clean-up standards for trace elements between countries: Why do they differ? J. Soil Sediment 2006 6 173 171

[6] De Sousa C Contaminated sites: The Canadian situation in an international context J. Environ. Manage 2001 62 131 154 11434028 10.1006/jema.2001.0431

[7] Weber O Scholz RW Bühlmann R Grasmück D Risk perception of heavy metal soil contamination and attitudes toward decontamination strategies Risk Anal 2001 21 967 977 11798130 10.1111/0272-4332.215165

[8] Provoost J Reijnders L Swartjes F Bronders J Carlon C D’Alessandro M Cornelis C Parameters causing variation between soil screening values and the effect of harmonization J. Soil Sediment 2008 8 298 311

[9] Toccalino PL Norman JE Health-based screening levels to evaluate U.S. Geological Survey ground water quality data Risk Anal 2006 26 1339 1348 17054535 10.1111/j.1539-6924.2006.00805.x

[10] Patterson BM Cohen E Prommer H Thomas DG Rhodes S McKinley AJ Origin of a mixed brominated ethene groundwater plume: contaminant degradation pathways and reactions Environ. Sci. Technol 2007 41 1352 1358 17593741 10.1021/es0615674

[11] DETR Circular 2/2000 Contaminated Land: Implement of Part IIA of the Environment Protection Act 1990 Available online: http://en.wikipedia.org/wiki/Environmental_Protection_Act_1990 (accessed on 4 October 2000).

[12] Taiwan EPA (Environmental Protection Administration) Soil and Groundwater Remediation Internet Available online: http://sgw.epa.gov.tw/public/En/index.htm (accessed on 4 October 2010).

[13] Römkens PF Guo HY Chu CL Liu TS Chiang CF Koopmans GF Characterization of soil heavy metal pools in paddy fields in Taiwan: chemical extraction and solid-solution partitioning J. Soil Sediment 2009 9 216 228

[14] Römkens PF Guo HY Chu CL Liu TS Chiang CF Koopmans GF Prediction of cadmium uptake by brown rice and derivation of soil-plant transfer models to improve soil protection guidelines Environ. Pollut 2009 157 2435 2444 19345457 10.1016/j.envpol.2009.03.009

[15] Chen ZS Heavy metal contamination of flooded soils, rice plants, and surface waters in Asia Biogeochemistry of Trace Metals Adriano DC Lewis Publishers Inc Boca Raton, FL, USA 1992 85 107

[16] USEPA Risk Assessment Guidance Vol. 1 Human Health Evaluation Manual, Part A Office of Emergency and Remedial Response Washington, DC, USA 1989

[17] USEPA Risk Assessment Guidance for Superfund Vol. 1 Human Health Evaluation Manual, Part E, Supplemental Guidance from Dermal Risk Assessment Office of Emergency and Remedial Response Washington, DC, USA 2004

[18] ASTM E2081–00 Standard Guide for Risk-Based Corrective Action ASTM International West Conshohocken, PA, USA 2000

[19] ASTM ASTM, E1739–95 Standard Guide for Risk-Based Corrective Action Applied at Petroleum Release Sites ASTM International West Conshohocken, PA, USA 1995

[20] He JY Zhu C Ren YF Yan YP Jiang D Genotypic variation in grain cadmium concentration of lowland rice J. Plant Nutr. Soil Sc 2006 169 711 716

[21] WHO Fifty-fifth Report of the Joint FAO/WHO Expert Committee on the Food Additives WHO Technical Report Series 901 World Health Organization Geneva, Switzerland 2001 61 69 97 98

[22] Hseu ZY Chen ZS Jien SH Pedological characteristics and heavy metals contamination of the paddy soils in Taiwan J. Integr. Field Sci 2007 4 41 64

[23] Nathanail CP Bardos RP Reclamation of Contaminated Land John Wiley & Sons West Sussex, UK 2008

[24] USEPA Child-specific Exposure Factors Handbook EPA-600-P-00-002B; National Center for Environmental Assessment Washington, DC, USA 2002

[25] USEPA Integrated Risk Information System (IRIS) Available online: http://www.epa.gov/iris/index.html (accessed on 4 October 2010).

[26] Miguel E Iribarren I Chacon E Ordonnez A Charlesworth S Risk-based evaluation of the exposure of children to trace elements in playgrounds in Madrid Spain Chemosphere 2007 66 505 513 16844191 10.1016/j.chemosphere.2006.05.065

[27] USEPA Exposure Factors Handbook: Volume I General Factors Office of Research and Development U.S. Environmental Protection Agency Washington, DC, USA 1997

[28] Jang CS Liu CW Lin KH Wang SW Spatial analysis of potential carcinogenic risks associated with ingesting arsenic in aquacultural tilapia (*Oreochromis mossambicus*) in blackfoot disease hyperendemic areas Environ. Sci. Technol 2006 40 1707 1713 16568791 10.1021/es051875m

[29] USEPA Risk-based Concentration Table U.S. Environmental Protection Agency Washington, DC, USA 1996

[30] Glass DJ Economic potential of phytoremediation Phytoremediation of Toxic Metals Raskin I Ensley B John Wiley & Sons New York, NY, USA 2000 15 31

